# Lay perspectives on social distancing and other official recommendations and regulations in the time of COVID-19: a qualitative study of social media posts

**DOI:** 10.1186/s12889-020-09079-5

**Published:** 2020-06-19

**Authors:** Sabahat Ölcer, Yüce Yilmaz-Aslan, Patrick Brzoska

**Affiliations:** 1 Health Services Research, Faculty of Health, School of Medicine, Witten/Herdecke University, Alfred-Herrhausen-Straße 50, 58448 Witten, Germany; 2 Department of Epidemiology & International Public Health, School of Public Health, Bielefeld University, 33501 Bielefeld, Germany

**Keywords:** Social distancing, Self-isolation, Coronavirus, COVID-19, SARS-CoV-2, Social media

## Abstract

**Background:**

COVID-19 caused by a new form of coronavirus (SARS-CoV-2) first appeared in China end of 2019 and quickly spread to all counties of the world. To slow down the spread of the virus and to limit the pressure on the health care systems, different regulations and recommendations have been implemented by authorities, comprising amongst others the closure of all entertainment venues and social distancing. These measures have received mixed reactions, particularly from young individuals, with many not following available advice. Drawing on the information in social media discussion forums, the present study explores the reasons why people ignore the orders and recommendations of the authorities and why the authorities are unable to produce a shared sense of inclusion concerning protective measures against the COVID-19 outbreak.

**Methods:**

Three open-access social media forums (Reddit, Twitter, and YouTube comments) were systematically searched with respect to COVID-19-related beliefs, attitudes, and behaviours of individuals. The data was retrieved in the first 3 weeks of March 2020. Qualitative document analysis and qualitative content analysis were used as the methodical approach. The data was reviewed by all authors and jointly interpreted to minimise inconsistencies.

**Results:**

The study reveals that reasons such as information pollution on social media, the persistence of uncertainty about the rapidly spreading virus, the impact of the social environment on the individual, and fear of unemployment associated with inequality in the distribution of income lead people to ignore the orders and recommendations of the authorities. The findings suggest that government representatives and politicians could not produce a shared sense of inclusion concerning protective measures against the COVID-19 outbreak, due to not building trust among the public and taking concrete economic steps to satisfy them.

**Conclusion:**

In uncertain crises, transparency in the presentation of information and government policies emerge as influential determinants in creating social susceptibility and solidarity. The differences between social classes constitute one of the important factors that affect the decision-making mechanisms of individuals in determining the necessary steps to be undertaken in times of crisis.

## Background

COVID-19 is a previously unknown condition that emerged in Wuhan, China’s Hubei province, in early December 2019. Considering the first symptoms, the disease was originally recorded as ‘a number of pneumonia cases of unknown origins’ [1]; however, at the end of the first week of January 2020, it was identified by the Chinese authorities as a new type of coronavirus [2] later termed Severe Acute Respiratory Syndrome Coronavirus 2 (SARS-CoV-2). SARS-CoV-2 has rapidly spread to other parts of China, then to neighbouring countries such as Thailand, Korea, and Japan before finally emerging in many other parts of the world [1]. Based on its sphere of influence and the growth in the number of cases, the World Health Organization (WHO) declared COVID-19 an outbreak on 11 March 2020 [3]. Until April 05, SARS-CoV-2 has spread to 210 countries and territories around the world, and as of 23 May 2020, over 5.1 million confirmed cases and almost more than 330.000 COVID-19-related deaths have been reported [4].

From the daily situation reports of the WHO [5], it becomes evident how COVID-19 evolved into a global crisis in every respect and how WHO’s public health strategic objectives have changed from simple to intricate measures. In parallel, basic protective measures taken by local and national authorities to slow down the spread of SARS-CoV-2, to limit the pressure on the health care systems, and hence to curb the number of COVID-19-related causalities have been implemented all over the world. They often began with limited measures on the local level and were soon extended to the closure of national borders and, finally, severe restrictions on everyday life. Sanctions such as the closure of hotels, restaurants and other entertainment venues, the cancellation of all public meetings, the closure of schools and universities, in some places the subsequent declaration of a curfew are examples of notable measures taken to reduce the incidence of SARS-CoV-2 infections. Aside from these structural measures, almost all national and regional governments in the world have issued different sets of recommendations or regulations, oftentimes implemented as decrees, on how its people should change their behaviour in order to decrease the risk of both contracting SARS-CoV-2 and passing it to others, especially those at particular risk for a severe course of COVID-19. For example, in Germany [6], aside from the recommendations on hygienic measures (particularly washing hands often), these decrees comprise the avoidance of close contacts (especially staying 6 feet or two meters apart from other people) (i.e., ‘social distancing’), including gathering of more than two persons, and in some federal states of Germany even encompass ‘contact bans’ (i.e., prohibiting the gathering of more than two persons, except those living together).

However, the protective precautions such as self-isolation, the rules on social distancing, and the closure of all entertainment venues in the public sphere have received mixed reactions, particularly from young individuals, many of which do not comply with current advice. For instance, beaches, hiking trails, and parks in California and Florida were swarmed with crowds over the weekends thus defying the bans and recommendations of authorities on social distancing [7]. In France, Belgium, England [8], the USA, Germany, and New Zealand [9], an increase in parties has been reported by the media, particularly after the closure of schools and universities. As a consequence, authorities frequently criticised their citizens for not complying with the official regulations and recommendations [9].

The videos and pictures illustrating non-compliance with social distancing recommendations and regulations, which occupied social media for days, have been accompanied by many discussions. Governments’ methods of coping with SARS-CoV-2, particularly imposing social distancing and self-isolation regulation, have led to a divide between people: the rule followers and the risk-takers [10]. The issues of human judgment, decision-making [11, 12], and personal freedom [13] in association with social conscience and solidarity and shared sense have been at the centre of the debates. Due to the opposition expressed by some individuals to the orders and recommendations of the authorities, discussions are still ongoing in order to understand perceptions, behaviours, attitudes, and reactions of individuals towards the current crisis.

Drawing upon the information in social media discussion forums, the present qualitative study explores the reasons why people ignore the instructions and recommendations of the authorities. It further explores, which actions guide their thinking when assessing the risks of SARS-CoV-2 and COVID-19, what their beliefs, attitudes and behaviours related to the COVID-19 outbreak are and why authorities are unable to produce a shared sense of inclusion concerning protective measures.

## Methods

Qualitative document analysis, which enables the evaluation of printed and electronic materials, was used to examine the social media posts. Documents included text and images obtained from the post [14].

### Selection of data

Three online discussion websites/forums (Reddit, Twitter, and YouTube comments) on social distancing, self-isolation, and COVID-19 were examined. The common ground of these websites is that they are widely used by the population. The target audience of this research is users who share the posts, including the reasons for not following the recommendations of the authorities regarding the COVID-19 outbreak. The data was retrieved in the first 3 weeks of March 2020. Posts and responses to the posts were examined one by one, and the data considered to be of relevance to the objective and research question of the study was copied to a text document. The posts were mostly in English, but in Twitter and YouTube forums, some German posts directly pertinent to the aim of the study were also encountered. Therefore, English and German posts were used in the study. German posts were translated into English before analysis.

### Data analysis

The data selected from the three social media forums differed in terms of the number of comments on threads, the content and types of comments, and the text written. The number of threads on Twitter was higher, posts contained more pictures or videos and, per the restrictions imposed by the service, were much shorter than in the other two social forums, and the texts included more emoji and slang words. Therefore, although the number of threads taken from Twitter is high, fewer threads were used for analysis. As the social distance topic was more recent in the social forums of YouTube and Reddit compared to Twitter, the number of threads was low, but the number of comments for each thread was high. Posts were textual, and the use of emoji and slang words on YouTube was higher than on Reddit. Texts on Twitter and YouTube included more topics on accusing authorities and politicians, distribution of income, and the impact of the social environment on the individual. The texts on Reddit also involved the uncertainty about the COVID-19 outbreak.

**Fig. 1:**
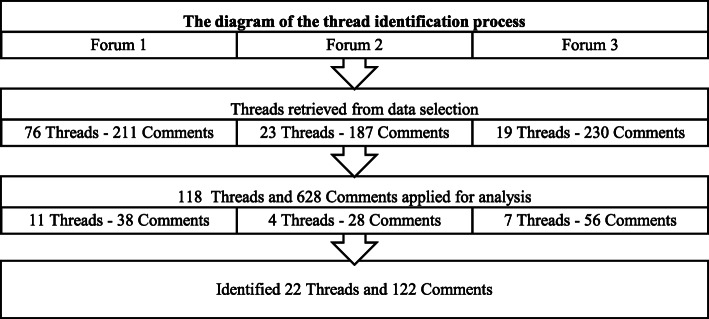
Illustration of the thread identification process

Posts copied to the text document were analysed using qualitative content analysis. The data were analysed manually due to the frequent use of abbreviations and “web speak” on these discussion forums [15]. Coding of the data arisen from the examinations of primary data was carried out with inductive content analysis. The data were systematically coded by one of the authors (SÖ). After that, categories and sub-categories were generated from the codes. Given the purpose and questions of the study, in addition, themes and sub-themes were created to increase the depth of analysis. The identified codes, categories, themes and sub-themes were then reviewed by all authors, differences were discussed, and themes and sub-themes were jointly interpreted to minimise inconsistencies. The thread identification process is illustrated in Fig. 1.

### Ethical issues

The posts in the discussion forums were selected from open boards; therefore, the user contributions were considered to be in public domain [16, 17]. For purposes of presentation in the present study, a standardised identifier (“user”) is used instead of the names and online identities of users in order to ensure their anonymity. In addition, verbatim quotations were not used. Also, identifiable details such as emending spelling errors and abbreviations were changed, thereby still preserving the meaning of the posts, due to the fact that posts can be obtained using a search engine, which would compromise anonymity [15, 18].

## Results

According to the users’ posts, six main themes and sub-themes were identified given the purpose and questions of the study.

### Information pollution on social media

Despite (or rather because of) uncertainty concerning SARS-CoV-2 and COVID-19, numerous interpretations and comments made in different areas and reinterpretation of information by second and third parties have led to ‘infollution’ (information pollution), misleading advice, and confusion on social media. This is illustrated in the following post:

*“What has given these people the permission to think that they know better than science and every single qualified authority on all matters?”* [User #2]

*“This is the result of a media that nobody can trust. I still do not know the truth.”* [User #77]

Questioning the source of available information connotes the need and expectation for qualified information from scientists and related authorities such as departments or ministries responsible for health. Apart from these sources, it can be asserted that other sources that provide selective and qualified information about the COVID-19 outbreak leave a similar impression on individuals, as explained by one of the users:

*“You have got to give it to Vox [American news and opinion website] for all these quality videos on this pandemic. Probably the most informative videos out there.”* [User #22]

According to the users’ posts, the information on social media that only the elderly belong to a high-risk group than young individuals has led to misunderstanding, as exemplified by the following users:

*“A dear friend in [X, referring to anywhere] is in a coma with coronavirus. He is under 40 and was in excellent condition [with a surprised expression].”* [User #54]

*“That notion of generational immunity is a fatal falsehood. There are steadily increasing, substantiated instances of typically ‘healthy’ adults in their 30s who became ill and are now on respirators. This has to be taken seriously by everyone, or no one is safe!”* [User #55]

This misunderstanding has induced an increase in beliefs among many people, particularly young individuals, that the measures taken and the regulations and recommendations emphasised by the authorities are not necessary and that the COVID-19 outbreak is exaggerated. This has also caused SARS-CoV-2 as such to be underrated by them.

### The need to know the unusual threat that spreads rapidly

The lack of experience with such an outbreak has led to confusion among the population. Despite the vast media coverage in light of an increasing number of cases, the health literacy in part seems to be low as the subsequent posts illustrate:

*“How can you spread a virus, if you are healthy and are not carrying it?”* [User #79]

*“But we do not even know how the virus is spread.”* [User #18]

The perplexity about the spread of SARS-CoV-2 and the symptoms of COVID-19 have gained depth on social media forums as a result of unmet information needs related to the outbreak. The following posts contain the discussion about the relationship of the spread of SARS-CoV-2 and the symptoms of COVID-19:

*“You do not even have to be just like her if you got something that starts spreading before symptoms develop. So you are spreading it before you even know you are sick. And unless I am mistaken, COVID-19 does spread before you have symptoms, so that it makes everyone that catches it a Typhoid Mary [referring to Mary Mallon] for a short while.”* [User #3]

*“Until now, it has only been foremost expressed that the virus is transferred via droplets and the mouth. It has then been said, less so, that the virus is contracted from surfaces, especially metallic surfaces, for up to three or four days. Coins are metallic. So why is there no campaign for getting people to clean their coins? Is not it just like soldiers marching all over America during the Spanish flu pandemic? […] Would not these little things help a lot?”* [User #23]

Both posts demonstrate that as uncertainties are reduced, particularly as exemplified in the second post, the debates on the COVID-19 outbreak change towards the precautions to be taken and the solutions to be developed.

### The impacts of the social environment

In some posts, the disobedience to protective precautions is discussed in the context of personal freedom. The users explain:

*“This is an attack on our rights; specifically, the right to peacefully assemble is being infringed.”* [User #52]

*“Yeah, sadly these people view a few weeks of stay-home order as a violation of their freedom and rights.”* [User #24]

This relationship has an influence on individuals’ decision-making processes and shows how current debates turn into motivational actions that guide thoughts of individuals, as also revealed by the chat of two users:

*“I just went to a crowded Red Robin [referring to a restaurant], and I am 30. It was delicious, and I took my sweet time eating my meal. Because this is America. And I will do what I want.”* [User #72]

*“The saddest part is that so many people get triggered by it; Red Robin is now the top trend on Twitter!”* [User #73]

The second post contains an interpretation associated with the first post. Interpretations and responses on social media forums indicate that pictures, videos or individuals sharing relevant activities arouse curiosity and/or find reciprocity among people.

### The role of the government’s representatives and politicians

#### Accusing authorities and politicians

According to the posts, the endeavours by the pertinent authorities for deceleration of the spread of the COVID-19 outbreak and protecting the public have not shown the expected sensitivity and solidarity in public, mainly due to the role that authorities and politicians played in the process. The criticisms mainly included the authorities and politicians in the United States and the United Kingdom:

*“Simple rules to live by during this Pandemic (COVID-19) 2020:*

*1. Assume you have the virus!*

*2. Stay away from everyone! 6 feet away!*

*3. Wash your hands with soap for 20 seconds frequently!*

*4. Stop touching your face! Wear gloves to remind you!*

*5. Blame [X, referring to authority]! Although he was warned about this in November 2019, he did not react until March 2020!*

*6. Remember that we have 17 spy/intelligence agencies around the world working 24/7 that keeps [X] and our government informed. [X] is saying, “He did not know” is just an outward LIE!”* [User #20]

*“The UK to the world: What is going on?*

*World: Take cover!*

*UK: From what? I do not see anything.*

*World: The virus!*

*UK: Ah, it will not hurt.”* [User #97]

Accusations made are further extended to blaming authorities for supposedly delaying the basic protective precautions and for not perceiving the COVID-19 outbreak as a serious threat from the beginning. In other words, the lack of common sense is associated with negligence by the authorities:

*“Coming from the UK, it is worrying to see how slowly the government are responding to this and is embarrassing that Londoners are still ignoring the outbreaks’ severity.”* [User #30]

*“Governments around the world have already killed the majority of us by not being proactive.”* [User #93]

#### The need for strong government actions and liability

Whilst some people have opposed the regulations and recommendations of the authorities, others have criticised the elected representatives for not sufficiently fighting against the COVID-19 outbreak by making tougher decisions. As explained by the users:

*“The Florida governor should have taken this [referring to coronavirus outbreak] seriously and should have closed the beach.”* [User #105]

*“Some people are dumb. Unless some kind of restrictions is made official, they will not listen or understand.”* [User #32]

Users highlight the need for strong government actions, such as mandatory actions taken in China and North Korea:

*“People like them [referring to people who defy the bans and recommendations of authorities on social distancing] justify the need for some strong government actions like the Chinese or North Koreans.”* [User #24]

#### Politicians’ conflict of interest

Politicians’ conflict of interest is considered to be one of the obstacles to appropriately focussing on and addressing the COVID-19 outbreak. On the one hand, it leads to a lack of confidence in politicians, especially their regulations and recommendations, and on the other hand, initiatives are not considered reasonable:

*“I hate how conservatives have turned a global pandemic into their little personal freedom and victimization tantrum.”* [User #5]

*“We absolutely […] resist government run amok taking advantage of a crisis. This is how your liberty dies. Stand up America and resist!”* [User #60]

In general, these posts show the pivotal role, which depending on the perspective is either positive or negative, that authorities and politicians have played in addressing the COVID-19 outbreak.

### Aids without concrete economic steps to satisfy them

#### Economy versus virus

Some posts reveal that people need to decide between ‘protect themselves for health reasons’ and ‘earn money to survive’. Decision-making and human judgment are affected by individual priorities that trigger people about what they need to do, as shown by the posts as follows:

*“If you are scared of getting sick, do not go outside. Wash your hands, etc. We need to start isolating the sick and at-risk people. Not everyone. Life and death are tied to the economy. Not just viruses.”* [User #62]

*“If you really want to protect Angelenos [referring to inhabitants of Los Angeles], freeze rent now! And if you really want people to stay home, protect those without housing. We can start by saving lives there.”* [User #81]

#### Fear of unemployment

Fear of unemployment can be handled with individual priorities, as it is one of the reasons that affect the decision-making process; however, the posts include references to the users’ own economic situations. According to the posts, people are concerned about their future due to economic uncertainty and expect solutions that involve different social classes:

*“I need the basic income now because I am currently applying for a visual impairment in the IT sector, and despite the alleged lack of skilled workers, no one is hiring.”* [User #69]

*“I know of too many people being threatened with unemployment if they do not attend work when they are not a key worker.”* [User #85]

*“What I will live on next month - I do not know!”* [User #68]

#### Neoliberal policies

Authorities are planning economic packages in order to protect companies and to provide economic stabilisation. Yet some posts suggest that economic recoveries will only be for large companies. People therefore expect concrete emergency programs covering different social classes. The subsequent posts exemplify the perception of individuals about economic measures taken by the authorities:

*“Everything that should be invested in the emergency program in the social area is blocking! The only help for business, banks and medicine are supported! Pure neoliberalism!”* [User #59]

*“... they will throw money like water at the rich to make sure they do not suffer.”* [User #8]

### Self-criticism by parents related to the behaviours and attitudes of their children

‘How did we raise our children?’ is the main question that gains prominence by parents after the disapproving reactions of young people to the basic protective measures taken by authorities. This is emphasised by users as follows:

*“These young people do not care about us, –old folks. We cannot blame them; we taught them to only care about their own personal happiness.”* [User #25]

*“... historically, youth has always had a sense of immortality which is compounded by the ‘screen’ addiction in 2020 [...] and which has been detrimental to any skills of real-world, self-reliance or community awareness that previous generations may have had. Plus, after carefree unsupervised childhoods [...], my generation and the next generation parents have become helicopter parents, creating co-dependency and entitlement - at the expense of civic or community duty.”* [User #51]

The reactions of young people to the precautions taken by the authorities can be explained by many reasons. However, according to the posts, the effect of the results of the use of technology stands out as the most obvious reason why social awareness cannot be created in the younger generation:

*“..., these are zoomers [referring to members of Generation Z, born in the late 1990s and early 2000s] [or] weaklings who grew up with technology and internet their entire lives, unlike the rest of humans […]”* [User #28]

Concerning the descriptions of parents mentioned above, their perception of the young generation is as follows:

*“Embarrassing! This is America’s younger generation.”* [User #101]

*“The final generation before the next big war; the calm before the storm.”* [User #29]

The posts show that the behaviours and attitudes of the young generation are considered socially unacceptable by the older generation and that a low level of expectations of parents from young individuals is intertwined with the rise in their levels of worries about the future.

## Discussion

By means of content posted to three social media forums, the present study examined the perspectives of individuals to the recommendations and regulations of authorities taken to address SARS-CoV-2 and the COVID-19 outbreak. In line with the declaration of the WHO [3], it can be observed that social media users perceive and accept the COVID-19 outbreak as a global crisis. However, users’ posts reveal two distinct groups, namely rule followers and risk-takers as defined by their reactions to the governments’ methods of addressing the COVID-19 outbreak [10]. They further show that due to the misperception that occurred on social media, two different groups have emerged based on the possibility of contracting SARS-CoV-2: the elderly as a high-risk group and young individuals as a lower risk group [19, 20].

With the increasing number of COVID-19 cases, authorities took different precautions against the outbreak. These measures, naturally, are not only restricted to authorities and departments or ministries but first and foremost require the participation of the public. Yet the posts point out that among some individuals these measures are perceived as an attack to personal freedom (see also the respective media perceptions: [10, 11, 13, 21, 22]) resulting in the consequence that human judgment and decision-making may be processed differently than expected. The lack of experience with such an outbreak has in some cases led to confusion among both the authorities and the public, which has been further affected by conflicting government messages. Hence, inconsistent measures taken late by some governments, information pollution on social media, the emergence of the risk-takers group as well as their prominence in social media and the public to some degree may be explained by the uncertainties related to a situation unknown in contemporary times.

The users’ posts show that there is a strong connection between shared sense/participation and transparency [23, 24]. The gradual increase of information and demystification of the COVID-19 outbreak has led to a decline in information pollution on social media; the debates on the forums have evolved towards the precautions to be taken and the solutions to be developed. In a similar vein, it can be observed that people who do not have in-depth knowledge about SARS-CoV-2 and COVID-19 tend to perceive precautions taken as an attack on their freedom. A survey by BVA-Doxa and Gallup conducted in mid-March 2020 on the link between the willingness to sacrifice some human rights and helping to prevent the spread of SARS-CoV-2 shows that large percentages of the populations in six European countries (the United Kingdom, the Netherlands, Germany, France, Italy, and Austria) support these measures. However, in Italy (93%), the Netherlands (91%), Austria (94%) and France (84%), where severe restrictions have been implemented from the very beginning of the COVID-19 outbreak, the acceptance rates are higher than in Germany (71%) and the United Kingdom (72%) [25].

The relationship mentioned above is supported by the users’ posts, which cover criticisms raised against authorities and politicians concerning the delay of the basic protective precautions taken and for not perceiving the outbreak as a severe threat from the very beginning. Some users have criticised authorities and their representatives for not sufficiently addressing the COVID-19 outbreak by means of tougher decisions. Furthermore, they have highlighted the need for strong government actions, citing mandatory actions taken in China. Asian countries/administrative regions, particularly Korea, Singapore, Hong Kong, and Taiwan, draw attention by users due to their attributed organisational skills and their experiences on how to deal with prior epidemic crises such as the severe acute respiratory syndrome (SARS) [26]. Germany can be another example in this regard, particularly in terms of the German chancellor’s perceived ability to manage the upheaval in recent weeks [27]. These cases demonstrate that transparency and perceived ability to manage crises enhance the feeling of confidence, and in parallel, they lead to an increase in a shared sense of inclusion [28].

In the present study, posts reveal that the impacts of the COVID-19 outbreak vary in different social classes and that people feel obliged to decide between ‘protect themselves for health reasons’ and ‘earn money to survive’, particularly in socially disadvantaged population groups. They also suggest that people are concerned about their future due to economic uncertainty and expect transparent solutions that include different social classes. In fact, authorities of almost all countries have announced economic packages to protect companies and to provide economic stabilisation. However, users’ posts suggest that the expressions of economic recovery did not create the expected impressions among the population because of the perception that economic programs are organised solely to protect large companies.

Based on the social media posts, it can be inferred that associated with uncertainties about the SARS-CoV-2 infection and conflicting messages on the internet, the social environment is another factor relevant to the disobedience to protective precautions taken because of its influence on individuals’ decision-making processes [29]. The reactions of young people to the basic protective measures taken by the authorities have caused parents to question themselves about the behaviours and attitudes of their children. In other words, the self-criticism by parents suggests that the behaviours and attitudes of the young generation are socially unacceptable. Yet the current findings selected from social media forums indicate that an increased level of worries about the future does not lead to a rise in expectations that parents have towards their children. The impact of the internet on everyday life stands out as the most obvious reason why social awareness cannot be created in the younger generation. In addition to the general characteristics of the period they live in, the lack of experience with such an outbreak [30] and information pollution on social media [31] can be considered further reasons.

To the best of our knowledge, this is the first study examining the lay perspectives on measures taken by authorities to address a pandemic that is unprecedented in contemporary times. Using social communication platforms that offer a wealth of unbiased data on selected and prioritised issues [32], it is therefore one of the first attempts to in-depthly explore the relationship of ignoring the instructions and recommendations of the authorities on the one hand and producing a shared sense of inclusion within a limited time frame on the other hand. Some limitations also need to be considered. Only three social media websites that are widely used by the population were chosen as data sources; therefore, posts on the boards may not represent other social media forums. The current study provides a comprehensive analysis of the COVID-19 outbreak concerning the reactions of people to the recommendations and regulations of the authorities. However, social media users are younger than the average population and also differ in other characteristics. It therefore remains unclear to what extent our results are transferable to other population groups [33]. Since the corresponding information is not available, it was not possible to include the demographic and socio-economic information of the users in the evaluation of the social media contributions. For that reason, also, no contrasting analyses of COVID-19-related beliefs, attitudes, and behaviours with respect to these variables were possible.

## Conclusion

The findings of the study contribute to the understanding of the public’s behaviour in the time of COVID-19 and future global health emergencies. Human judgment, decision-making, and personal freedom in association with social conscience and shared sense were discussed as relevant issues to be considered in addressing global crises. The study reveals that transparency and a consistent approach play a key role in the development of participation-oriented measures to address global crises such as SARS-CoV-2/COVID-19. The differences between social classes constitute one of the important factors that affect the decision-making mechanisms of individuals in determining the steps to be taken in times of crisis. Future research should be related to how and in what ways the COVID-19 outbreak affects individuals with chronic disease. It should further explore how the impact of the lack of transparency and a consistent approach psychologically affects individuals during the COVID-19 outbreak. The study, in this respect, highlights the importance of presenting qualified information on social media, manifesting its advantages and disadvantages. The results further suggest the potential advantages and opportunities of using social media data in scientific investigations.

## Data Availability

All posts used for the analysis are available from the corresponding author upon request.

## Abbreviations

COVID-19: Coronavirus disease 2019
ORB: Opinion Research Business
SARS: Severe acute respiratory syndrome
SARS-CoV-2: Severe acute respiratory syndrome coronavirus 2
WHO: World Health Organization
